# Amendment of the OMERACT ultrasound definitions of joints’ features in healthy children when using the DOPPLER technique

**DOI:** 10.1186/s12969-018-0240-2

**Published:** 2018-04-10

**Authors:** P. Collado, D. Windschall, J. Vojinovic, S. Magni-Manzoni, P. Balint, G. A. W. Bruyn, C. Hernandez-Diaz, J. C. Nieto, V. Ravagnani, N. Tzaribachev, A. Iagnocco, M. A. D’Agostino, E. Naredo, on behalf of the OMERACT ultrasound subtask force on pediatric

**Affiliations:** 1 Hospital Universitario Severo Ochoa., Madrid, Spain; 2 Department of Pediatrics, Asklepios Hospital Weissenfels, Weissenfels, Germany; 3 Department of Pediatrics, Clinical Center, Faculty of Medicine, University of Nis, Nis, Serbia; 4 Rheumatology Division, IRCCS Ospedale Pediatrico Bambino Gesù, Rome, Italy; 5 3rd Department of Rheumatology, National Institute of Rheumatology and Physiotherapy, Budapest, Hungary; 6 Department of Rheumatology, MC Groep, Lelystad, the Netherlands; 7 Instituto Nacional de Rehabilitación, Mexico City, Mexico; 8 Department of Rheumatology, Hospital Universitario Gregorio Marañon, Madrid, Spain; 9 Department of Internal Medicine, ASST Mantova, C. Poma Hospital, Mantova, Italy; 10 Pediatric Rheumatology Research Institute, Bad Bramstedt, Germany; 11 Rheumatology Unit, Sapienza Università di Roma, Rome, Italy; 12 Rheumatology Department, Hôspital Ambroise Paré, Boulogne Billancourt; INSERM U1173, Laboratoire d’Excellence INFLAMEX, UFR Simone Veil, Versailles-Saint-Quentin University, Yvelines, France; 13 Department of Rheumatology, Hospital Universitario Fundación Jimenez Díaz and Autonoma University, Madrid, Spain

**Keywords:** Ultrasonography, Power-Doppler, Pediatric rheumatology, Joint anatomy

## Abstract

**Background:**

Recently preliminary ultrasonography (US) definitions, in B mode, for normal components of pediatric joints have been developed by the OMERACT US group. The aim of the current study was to include Doppler findings in the evaluation and definition of normal joint features that can be visualized in healthy children at different age groups.

**Methods:**

A multistep approach was used. Firstly, new additional definitions of joint components were proposed during an expert meeting. In the second step, these definitions, along with the preliminary B-mode-US definitions, were tested for feasibility in an exercise in healthy children at different age groups. In the last step, a larger panel of US experts were invited to join a web-based consensus process in order to approve the developed definitions using the Delphi methodology. A Likert scale of 1–5 was used to assess agreement.

**Results:**

Physiological vascularity and fat pad tissue were identified and tested as two additional joint components in healthy children. Since physiological vascularity changes over the time in the growing skeleton, the final definition of Doppler findings comprised separate statements instead of a single full definition. A total of seven statements was developed and included in a written Delphi questionnaire to define and validate the new components. The final definitions for fat pad and physiological vascularity agreed by the group of experts reached 92.9% and 100% agreement respectively in a web survey.

**Conclusion:**

The inclusion of these two additional joints components which are linked to detection of Doppler signal in pediatric healthy joints will improve the identification of abnormalities in children with joint pathologies.

**Electronic supplementary material:**

The online version of this article (10.1186/s12969-018-0240-2) contains supplementary material, which is available to authorized users.

## Background

**Fig. 1:**
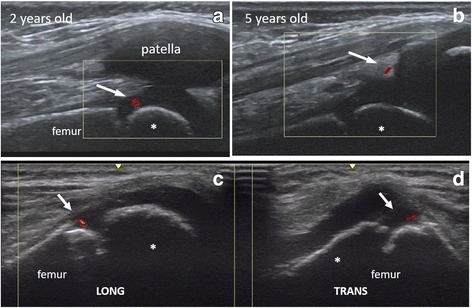
US images show normal anatomy of the knee joint. The upper images (**a**, **b**). We see the distal epiphyseal end of the femur with its secondary ossification centre (asterisk). Note a physiological vessel (arrow) in the epiphyseal cartilage of the femoral condyle (**a**) and in the quadriceps fat pad (**b**). Every vessel should be proved in longitudinal (**c**) and transverse view (**d**)

Musculoskeletal ultrasonography (US) has been shown to be a reliable, widely available, and child-friendly technique in the routine practice, particularly to detect joint inflammation in children with juvenile idiopathic arthritis (JIA) [1–5]. In adults with rheumatoid arthritis, both grey-scale (GS) and power Doppler (PD) US have been shown to be sensitive to change and predictive of developing arthritis and radiographic structural damage [6]. However, despite its great utility in adults, a systematic review concluded that the basis for the use of US in pediatric rheumatology has not been established yet [7]. In fact, the review showed that very few publications collected information on PD US. Given the unique anatomy of the growing child, it was not surprising that questions related to the presence of joint Doppler signals in the pediatric population were more difficult to answer than in adults. Moreover, several studies have shown that Doppler signal within joints is detectable in healthy children [8, 9]. To understand it, we have to take into account that normal growth and ossification in the developing skeleton is intimately related to the vascularity of the unossified epiphyseal and physeal cartilage [10–14] (Fig. 1).

The Outcome Measures in Rheumatology (OMERACT) US Pediatric Task Force was formed to standardize the use of US in juvenile arhritis. It has developed preliminary B-mode US definitions (or GS-US definitions) for joint components in healthy children [15], as well as, a standardized US scanning procedure specifically adapted to children [9]. However, vascularity of the unossified epiphyseal and physeal cartilage has not been described in detail yet [15]. At that point, some obstacles have been found (i.e., the features of this imaging modality, operator-dependent on expert and equipment, and the unclear terminology that is used to refer to physis, also known as “the growth plate” or “the epiphyseal growth plate”) [10–15].

**Fig. 2:**
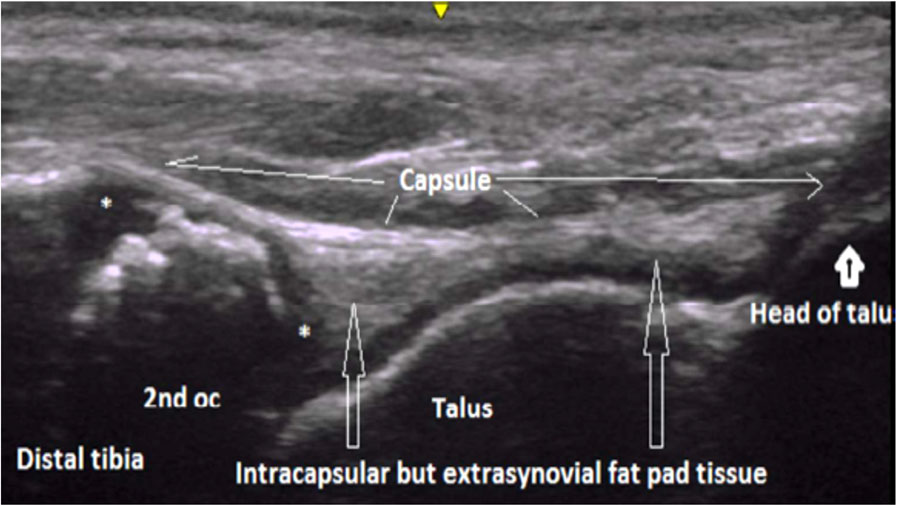
Longitudinal dorsal view of a healthy ankle joint. The US image shows the location of intracapsular but extrasynovial fatty tissue, the presence of physis and epiphyseal cartilage in distal end of the tibia (*). 2nd oc: secondary ossification centre of the tibia

Besides unossified cartilage, fat pads, particularly in the knee and ankle joints, can show Doppler signal (physiological vascularity). Small masses of fat are enveloped by the fibers of the joint capsule, which separate the fat pads from the synovial lining, making the fat pads intracapsular and extrasynovial in location. This anatomic arrangement is the basis for understanding the role of fat pad in development of inflammation at joint level [16, 17] (Fig. 2).

To describe the vascularity in healthy joints and develop its definition to add to the preliminary B-mode US definitions of joint components, would therefore improve the performance of US as an outcome measure in JIA clinical trials.

## Methods

### Study design

The new additional definitions were developed through a Delphi process involving three steps (Additional file 1 shows the workflow outlining the consensus process to develop and validate the new additional definitions). It was based on the OMERACT methodology previously described [15].

The first step was a face-to-face meeting among ten US experts in Germany. Most of them have been involved in development of the previous B-mode US definitions [15]. The aim was to identify the relevant joint components showing Doppler signal and the terminology used to describe them with the objective to use the results of this first step to draft actual definitions. The group reviewed and discussed a set of US images from pediatric healthy joints and literature review focusing on the vascularity of the unossified epiphyseal cartilage and fat pad [9–14, 18–20]. This set comprised 40 representative US images (12 knees, 12 ankles, 10 wrists, 6 metacarpophalangeal joints) of variously aged children from previous studies [9]. Several questions regarding PD findings located in the unossified epiphyseal and physeal cartilage and fat pad of the joint were discussed in order to plan the Delphi process. While ephyseal cartilage can be detected in the four joints, finger joint does not contain fat pad. Given that the secondary ossification centre fills in and replaces epiphyseal cartilage in bone throughout the growth of child, the process of ossification in the joint was analysed because of its relevance in the definition of the PD findings detected. The second step was to test the applicability of the previous B-mode and new PD-mode proposed US definitions (i.e., non-ossified hyaline cartilage, ossification center, joint capsule, synovial membrane, ossified bone, fat pad tissue, intra-articular vascularity) in an exercise involving 12 healthy children. The same ten US experts previously involved in the step 1 participated in the workshop. All experts were experienced (variable experience from 2 to 15 years) in musculoskeletal US in children. Four joints (i.e. knee, ankle, wrist, and second metacarpophalangeal (MCP II) joints) were examined in four different age groups (toddler and preschool ages 2–4 years, young children ages 5–8 years, preadolescent ages 9–12 years, and teenager ages 13–16 years) following standardized image acquisition and machine setting protocols [9]. Joint selection was based on previous studies and its common involvement in JIA [5, 9, 15, 20, 21]. The study was approved by the Ethics Committee of the Medical Board of Saxony-Anhalt (43/15) in Halle, Germany and was conducted in compliance with the Helsinki Agreement. Written consent was obtained from all parents and children prior to the exercise.

The examinations were performed on the same day, in the same room, using three different machines but identical brand (Logiq E, Logiq S8 and Logiq E9; General Electric Medical Systems, Waukesha, WI, USA) equipped with a 5–13 MHz broadband linear array transducer in the Logiq E and a 4–15 MHz broadband linear array transducer in the Logiq S8 and Logiq E9. PD was selected instead of colour Doppler based on the experience and daily use of the experts. The machines were calibrated with identical B-mode settings (frequency of 10–15 MHZ), but PD setting was optimized in the three US machine adjusting for the knee joint, wrist and tibiotalar joints, and MCP II joint as follows: pulse repetition frequency [PRF] 0.6, 0.8 and 0.8 MHZ respectively, and Doppler frequency [DF] 5, 7 and 10 MHZ respectively. It was emphasised the importance of correct size of the Doppler box, i.e. the Doppler box had to include the relevant joint structures and extend to the top of the image.

For each joint, participants were asked to assess the applicability of US definition (definition applicable: 1, yes or 0, not) attributed to each of joint components. Each participant evaluated real US images for applicability according to the following quality parameters (i) an image with appropriate magnification of the target structures and (ii) correctly displaying the wording of that relevant structure developed by the group consensus. The results were recorded on a preprinted data collection sheet.

The final step was to formulate a proposal wording of the new definitions considering the results obtained in the two previous steps in order to be presented to a larger panel of experts. Sixteen international experts were invited to join a web-based consensus process in order to reach consensus on the proposed definitions using the Delphi methodology. Most experts had participated in tasks of validation of US in adults or in children within the OMERACT US group at some time. The first online questionnaire comprised seven statements regarding definitions of interest. Expert agreement for each statement was rated using a 1–5 Likert scale [15]. If successive rounds were needed, it would include the statement that required modifications according to participant suggestions during the previous round interaction.

### Statistical analysis

Statistical analysis was performed using the software package SPSS, version 22 (IBM, Armonk, US) and the software package R (R Foundation for Statistical Computing, Austria). Applicability of the US definitions on each joint was calculated as the percentage of rates that scored it as yes (percentage of agreement on the applicability).

In the web based consensus process, the agreement was scored using a 1–5 Likert scale as follows: 1 = strongly disagree, 2 = disagree, 3 = neutral, 4 = agree, and 5 = strongly agree. An agreement ≥80% was considered mandatory for accepting the definition as appropriated.

## Results

### Step 1. Nominal group consensus

**Fig. 3:**
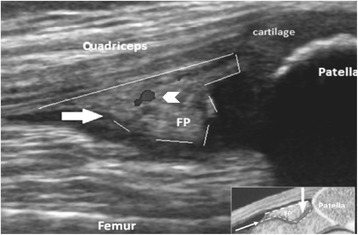
Midsagittal plane of the knee joint a 5-year-old healthy boy. The image shows physiologic joint vascularity (arrow head) located in the quadriceps fat pad (arrow)

Comments of the group showed the challenge to define physiological vascularity (normal Doppler signal) as a single item. Particularly applied to the epiphyseal cartilage and the ossification centre, which undergo significant changes throughout the maturation of the child. Therefore, they had to do a more cautious wording in this new definition. The new proposed US definitions for Doppler features were: 1) Doppler signal within the pediatric healthy joint can be detected as physiological vascularity of the unossified epiphyseal and physeal cartilage and the fat pads at any age during the growth, but since synovial membane is undetectable under normal circumstances, any Doppler signal observed in a synovial thickening should be considered an abnormality, 2) fat pad tissue can be present as an intraarticular structure, in a proper anatomical location, with heterogeneous echogenicity (similar to the US appearance of fat in the tissues below the skin) which might show Doppler signal (Fig. 3). Epiphyseal cartilage was not defined in this step, since the hyaline cartilage and the epiphyseal secondary ossification centre were included in the preliminary B-mode definitions.

### Step 2. Practical exercise

The applicability of the preliminary and new US definitions were assessed on a total of 48 joints within the different age groups by all 10 participants. A perfect agreement (100%) was reached on the preliminary GS-US definitions (i.e., the hyaline cartilage, the epiphyseal secondary ossification centre, the normal joint capsule, the normal synovial membrane, the ossified portion of articular bone) for each joint. The percentage agreement was 100% in the definition for fat pad for each joint, whereas agreement in physiological vascularity was variable depending on the kind of joint. A 100% agreement was reached in the wrist, but it was lower in the rest of joints. It was a 69.5% [mean 69.5%; range 67%–72%] in the MCP II, 83.3% [range 83.1%–88%] in the knee and 86.1% [range 83%–89%] in the tibiotalar joint.

### Step 3. Delphi consensus results

Taking into account the results of the practical exercise, definitions were reworded. It was suggested to provide separate statements instead of a full definition in order to identify components that would require further modification before their validation. Seven statements were therefore circulated to the panel of experts. The web based consensus process involved two rounds. In the second round the final version of the definitions obtained approval. This final version is shown in Table 1.

**Table 1:** Final statements and group agreement (percentage agreement) achieved for each statement of the second round

N.	Definition Statement	Percentage agreement, %
1	Physiological vascularity can be detected by PD as Doppler signal in the joint structures at any age during growth	100
2	Physiological intraarticular vascularity can be detected in children within the fat pads and unossified joint structures (i.e., the physis, the cartilage of epiphysis and the short bones cartilage)	85.7
3	Detection of physiological vascularity and its intraarticular anatomical position is joint and age (particularly in the youngest children) dependent	85.7
4	Physis can be detected in children as an anechoic unossified structure, intra- or extra-articular according to its anatomical location	85.7
5	Fat pad can be detected as an intra-articular structure with heterogeneous echotexture (similar to the subcutaneous tissue) which might show vascularity	92.9
6	In different age groups of children, due to the skeletal development, ossification centers can be detected with different maturation state	100
7	Ossification grade is age and joint dependent	92.9

All invited participants responded to the first and the second round of the Delphi questionnaire (100% response rate). In the first round, consensus for fat pad and physiological joint vascularity (92.9% and 100%, respectively) was reached (Table 1). Nevertheless, as the challenge for scanning children is to ensure the general applicability (i.e., the wide age range and all kind of joints), aiming to avoid specific age descriptions, the final wording for the definition of joint vascularity was adjusted to be applicable to all age ranges. Hence, the actual definition for sonographic features of physiological vascularity comprises several statements (i.e., statements 1–4).

Due to PD US was not used in preliminary definitions of joint components, physis was not described in detail [15]. Physis is a relevant structure that might show Doppler signal, but physis is not detectable in the fully ossified bone of an adolescent. One statement related to physis was included in the survey too. No consensus was found for physis in the first round (57%), but it was reached in the second (85.7%) when the statement was reworded clarifing that this structure could be intra- or extra-articular according to its anatomical location (Table 1). Similarly, the agreement for statement 3 was low in the first round (50%), but could exceed the threshold of 80% when the statement was rephrased. Here, the panel of experts suggested to clarify that detection and anatomical location of Doppler signal are mostly age dependent (Table 1).

Despite the ossification process was not considered as a joint component to define, its description was included in the survey. The statement number 6 and 7 are related to the ossification and reached almost 100% of agreement in the first round.

## Discussion

Without clear definitions of physiologic Doppler US findings in normal joints of children, it will be difficult to discriminate minimal active arthritis from physiologic growth patterns of joints. The interpretation of the Doppler signal in the pediatric joint is still being the most challenging and the least researched joint component.

We have used Doppler US to complete definitions of joints’ features in healthy children. We have successfully developed, tested and validated two additional definitions through the Delphi method used in previous OMERACT studies.

Despite the OMERACT US Pediatric Task Force has been working on definitions of B-mode US findings of the healthy joint [15], standardization of US scanning in children, and definitions for the US appearance of synovitis in children [22], none of those studies included systematically a definition of normal Doppler signal. Indeed, the main concern showed in the development of the definition of synovitis was the difficulty on how to define abnormal Doppler signal.

Because of the limitations identified in the previous studies [15, 22], our research activity started with a face to face meeting that permitted to clarify several issues with regard to what joint components and anatomical locations of Doppler signals should be considered taking into account the preliminary five definitions (the hyaline cartilage, the epiphyseal secondary ossification centre, the normal joint capsule, the normal synovial membrane, the ossified portion of articular bone) [15]. Besides the Doppler signal, it suggested to include the description of fat pad and physis in the present study in which Doppler signal might be detected and they were not define previously.

The practical exercise permitted the assessment of potential variation in image acquisition. As expected, we found a very good applicability of definition between experts when applying the preliminary OMERACT definitions for GS-US (namely, B-mode US) joint components. These findings enhance the results of previous study [15]. The disagreement between experts was related to the applicability of definition for Doppler signal; we found a good but variable agreement in all joints except for MCP joint. The disagreement for MCP was not later discussed during the consensus process, hence further investigation is needed. We are aware that several factors might contributed to that variability. First, as different PD settings were used depending on machine and joint, the impact of machines on the results could not have been minimised enough. Second, patient factors such as the small size of the MCP joint with slow blood flow velocity and restless children, may contribute to a lesser accurate assessment.

**Fig. 4:**
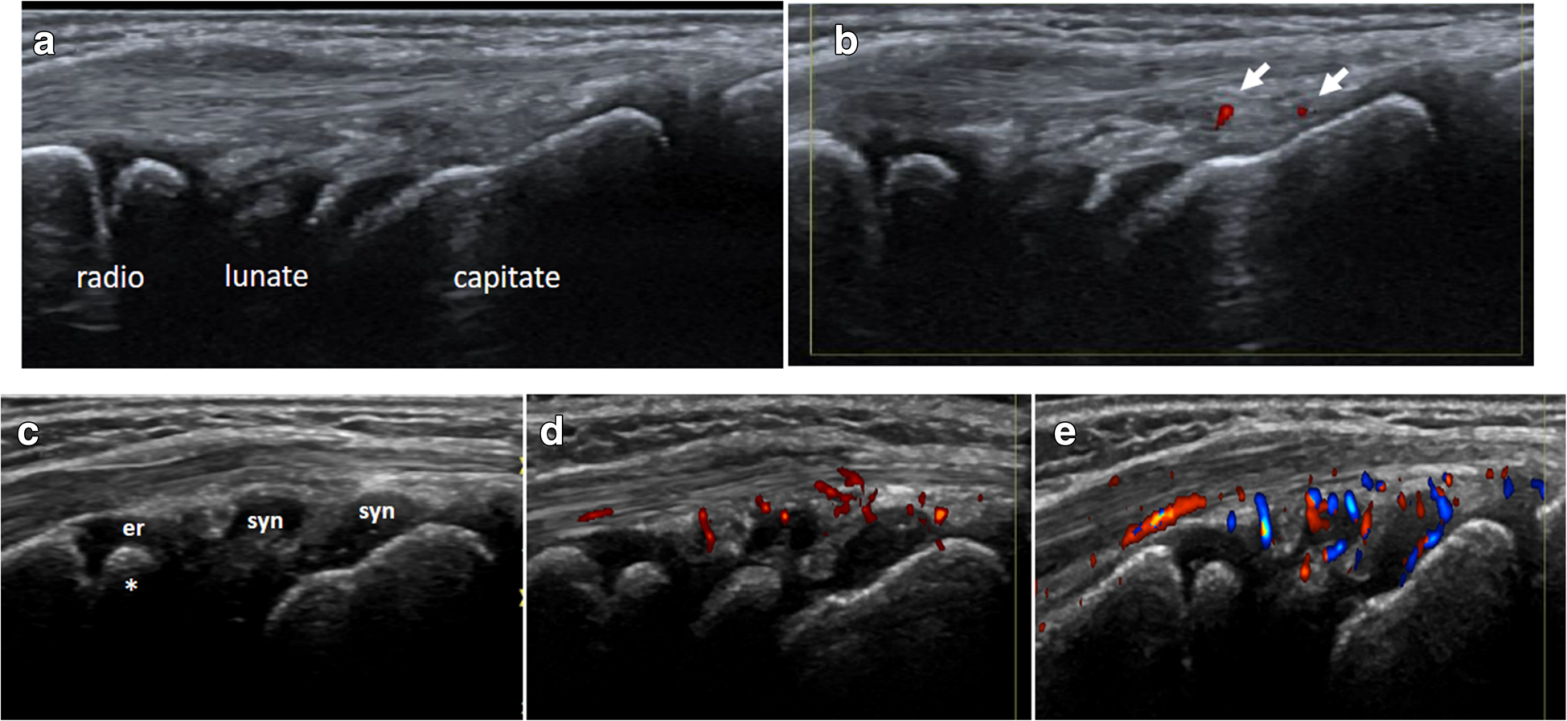
Longitudinal view of the dorsal aspect of the wrist joint in a 6-year-old child. The upper images (**a**, **b**) show the normal sonoanatomy on Grey-scale US (**a**) and power-Doppler US (**b**) showing single vessels close to os capitate. The lower images (**c**, **d**, **e**) show synovitis of the wrist joint in a patient with JIA. Synovial vascularity detected in the joint recesses by Doppler (image **d**, power Doppler and image **e**, color Doppler) reflects active inflammation. The distal epiphyseal cartilage of radius (er) is visible as an anechoic structure surrounding the secondary ossification nucleus (*). Dynamic examination let distinguish the epiphyseal cartilage of radius from effusion/synovitis (syn)

Doppler signal within joint or peri-articular is a source of uncertainty or misinterpretation for pediatric rheumatologist who infrequently perform the musculoskeletal US (Fig. 4). In addition, taking into account unclear meaning of Doppler signals in asymptomatic joints of children having JIA, it seems mandatory to include Doppler mode in the definitions of pediatric joint in order to enhance the validity of US in children [7, 23–25].

Seven separate statements were provide instead of a full definition, and most of them regarding physiological vascularity as a reflexion of its complexity. This complexity might be explained by (i) the availability of only a basic knowledge in Doppler findings in healthy children [9], (ii) a scarce validation in Doppler US assessment of pediatric joints [7] and (iii) the difficulty in the interpretation of the Doppler findings in children, particularly at the level of cartilaginous structures [26].

Two rounds of the Delphi exercise were needed to reach an agreement on US definitions of these additional joint components. Because of the fat pad tissue has already been considered in adults [27], its definition reached the agreement easier than expected. Nevertheless, a recent reliability study on MRI in JIA showed some discrepancies in the assessment of the knee fat pads [28].

Despite the fact that ossification is not a structural component of joint, we had to describe it for two important reasons: first, because bone landmarks are important for proper image acquisition during US scanning and second, because the appearance of physeal and epiphyseal cartilages changes through childhood; indeed, the physis (or the growth plate) should be considered in the oldest children as the unique cartilaginous structure of the growing skeleton which is displayed on US as an anechoic gap in the bone cortex [15].

Using the Doppler modality, we have produced additional US definitions for joint components that should be used in combination with the five published previously. Despite the present study represents an essential step toward a more reliable use of the Doppler technique in children, the Delphi approach has showed that the issue regarding physiological vascularity (i.e., normal Doppler signal) requires further investigations. Besides the expertise of sonographer, the potential effect of transducer pressure in small pediatric joints and the variation in Doppler sensitivity in different machines [29], may influence on acquiring of images and make difficult to provide a unique definition of the physiological vascularity. Since the aim of the study was to define components of the healthy joint as displayed on Doppler US, none validation of these definitions using a comparison imaging technique was done.

Our results are in line with others studies that show how the Doppler US can detect early inflammatory lesions and display an enhancement of physiological joint vascularity in JIA patients [30], and it can also show a increased physiological blood flow adjacent to the distal metaphysis and epiphysis of a long bone in acute osteomyelitis [31].

Although these definitions should be considered when US is applied on children with arthritis in daily practice, further studies are required to evaluate the applicability of these new PD definitions to other joints and to explore their potential use in clinical trials.

## Conclusion

The inclusion of these two additional joints components which are linked to detection of Doppler signal in pediatric healthy joints will improve the identification of joint abnormalities in pediatric rheumatic diseases.

## Additional file

Additional file 1: Workflow outlining the consensus process to develop and validate the new additional definitions. (TIFF 164 kb)

## Abbreviations

DF: Doppler frequency
GS: Grey-scale
JIA: juvenile idiopathic arthritis
MCP II: second metacarpophalangeal
OMERACT: Outcome Measures in Rheumatology
PD: Power Doppler
PRF: Pulse repetition frequency
US: Ultrasonography
