# Supplementary figures and images for: Amendment of the OMERACT ultrasound definitions of joints’ features in healthy children when using the DOPPLER technique
Source: Pediatr Rheumatol Online J. 2018 Apr 10;16:23. doi: 10.1186/s12969-018-0240-2 (PMC5892017; doi:10.1186/s12969-018-0240-2)

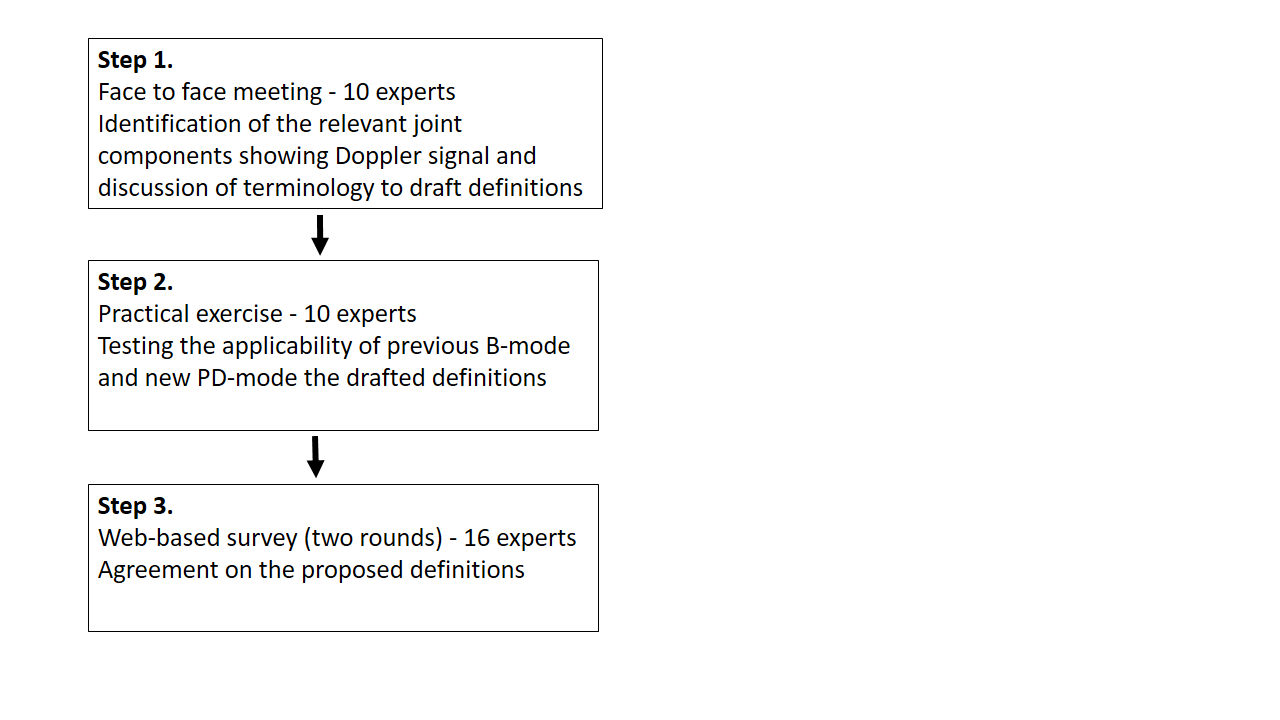

Supplement: Supplementary file 1 — Workflow outlining the consensus process to develop and validate the new additional definitions. (TIFF 164 kb) [file 12969_2018_240_MOESM1_ESM.tif]
